# Emergency department use and Artificial Intelligence in Pelotas: design and baseline results

**DOI:** 10.1590/1980-549720230021

**Published:** 2023-03-10

**Authors:** Felipe Mendes Delpino, Lílian Munhoz Figueiredo, Ândria Krolow Costa, Ioná Carreno, Luan Nascimento da Silva, Alana Duarte Flores, Milena Afonso Pinheiro, Eloisa Porciúncula da Silva, Gabriela Ávila Marques, Mirelle de Oliveira Saes, Suele Manjourany Silva Duro, Luiz Augusto Facchini, João Ricardo Nickenig Vissoci, Thaynã Ramos Flores, Flávio Fernando Demarco, Cauane Blumenberg, Alexandre Dias Porto Chiavegatto, Inácio Crochemore da Silva, Sandro Rodrigues Batista, Ricardo Alexandre Arcêncio, Bruno Pereira Nunes

**Affiliations:** Universidade Federal de Pelotas – Pelotas (RS), Brazil.; Duke University School of Medicine – Durham (NC), United States.; Universidade de São Paulo – São Paulo (SP), Brazil.; Universidade Federal de Goias – Goiânia (GO), Brazil.; Universidade de São Paulo – Ribeirão Preto (SP), Brazil.

**Keywords:** Machine learning, Chronic diseases, Multimorbidity, Urgent and emergency care, Aprendizado de máquina, Doenças crônicas, Multimorbidade, Urgência e emergência

## Abstract

**Objetivo:**

To describe the initial baseline results of a population-based study, as well as a protocol in order to evaluate the performance of different machine learning algorithms with the objective of predicting the demand for urgent and emergency services in a representative sample of adults from the urban area of Pelotas, Southern Brazil.

**Methods:**

The study is entitled “Emergency department use and Artificial Intelligence in PELOTAS (RS) (EAI PELOTAS)” (https://wp.ufpel.edu.br/eaipelotas/). Between September and December 2021, a baseline was carried out with participants. A follow-up was planned to be conducted after 12 months in order to assess the use of urgent and emergency services in the last year. Afterwards, machine learning algorithms will be tested to predict the use of urgent and emergency services over one year.

**Results:**

In total, 5,722 participants answered the survey, mostly females (66.8%), with an average age of 50.3 years. The mean number of household people was 2.6. Most of the sample has white skin color and incomplete elementary school or less. Around 30% of the sample has obesity, 14% diabetes, and 39% hypertension.

**Conclusion:**

The present paper presented a protocol describing the steps that were and will be taken to produce a model capable of predicting the demand for urgent and emergency services in one year among residents of Pelotas, in Rio Grande do Sul state.

## RESUMO

**Objetivo:**

Descrever os resultados iniciais da linha de base de um estudo de base populacional, bem como um protocolo para avaliar o desempenho de diferentes algoritmos de aprendizado de máquina, com o objetivo de predizer a demanda de serviços de urgência e emergência em uma amostra representativa de adultos da zona urbana de Pelotas, no Sul do Brasil.

**Métodos:**

O estudo intitula-se “Emergency department use and Artificial Intelligence in PELOTAS (RS) (EAI PELOTAS)” (https://wp.ufpel.edu.br/eaipelotas/). Entre setembro e dezembro de 2021, foi realizada uma linha de base com os participantes. Está previsto um acompanhamento após 12 meses para avaliar a utilização de serviços de urgência e emergência no último ano. Em seguida, serão testados algoritmos de *machine learning* para predizer a utilização de serviços de urgência e emergência no período de um ano.

**Resultados:**

No total, 5.722 participantes responderam à pesquisa, a maioria do sexo feminino (66,8%), com idade média de 50,3 anos. O número médio de pessoas no domicílio foi de 2,6. A maioria da amostra tem cor da pele branca e ensino fundamental incompleto ou menos. Cerca de 30% da amostra estava com obesidade, 14% com diabetes e 39% eram hipertensos.

**Conclusão:**

O presente trabalho apresentou um protocolo descrevendo as etapas que foram e serão tomadas para a produção de um modelo capaz de prever a demanda por serviços de urgência e emergência em um ano entre moradores de Pelotas, no estado do Rio Grande do Sul.

## INTRODUCTION

Chronic diseases affect a large part of the population of adults and older adults, leading these individuals to seek urgent and emergency care. The implementation in 1988 of the Unified Health System (SUS) resulted in a model aimed at prevention and health promotion actions based on collective activities^1^ – starting at Basic Health Units (UBS). There is also the National Emergency Care Policy, which advanced in the construction of the SUS, and has as guidelines universality, integrity, decentralization, and social participation, alongside humanization, the right of every citizen^2^.

In a study that evaluated the characteristics of users of primary health care services in a Brazilian urban-representative sample, it was found that the vast majority were women and part of poorer individuals, in addition to almost 1/4 of the sample receiving the national income distribution program (family allowance)^3^. Brazil is a country highly unequal in socioeconomic terms; approximately 75% of the Brazilian population uses the SUS and depends exclusively on it, and do not have private health insurance^4,5^. 

Individuals with multimorbidity are part of the vast majority who seek urgent and emergency services^6^. Multimorbidity is a condition that affects a large part of the population^7^, especially older adults^7^. In addition, the association of multimorbidity with higher demand for emergency services is a challenge to appropriately manage and prevent these problems^8,9^. 

Innovative approaches may allow health professionals to provide direct care to individuals who are more likely to seek urgent and emergency services. The use of artificial intelligence can make it possible to identify and monitor a group of individuals with a higher probability of developing multimorbidity. In this context, machine learning (ML), an application of artificial intelligence, is a promising and feasible tool to be used on large scale to identify these population subgroups. Some previous studies have demonstrated that ML models can predict the demand for urgent and emergency services^10,11^. Besides, a systematic review showed that ML could accurately predict the triage of patients entering emergency care^12^. However, in a search for studies in Brazil, we found no published article on the subject.

In Brazil, urgent and emergency services are a fundamental part of the health care network, ensuring timely care in cases of risk to individuals’ lives^9^. Urgent and emergency services are characterized by overcrowding and high demand. In addition, with the current pandemic of COVID-19, updated evidence on the characteristics of the users seeking these services is timely and necessary. The objective of this article was to describe the initial baseline results of a population-based study, as well as a protocol in order to evaluate the performance of different ML algorithms with the objective of predicting the demand for urgent and emergency services in a representative sample of adults from the urban area of Pelotas.

## METHODS

The present cohort study is entitled “Emergency department use and Artificial Intelligence in PELOTAS-RS (EAI PELOTAS)” (https://wp.ufpel.edu.br/eaipelotas/). The baseline was conducted between September and December 2021, and a follow-up was planned to be conducted 12 months later. We utilized the cross-sectional study to measure the prevalence of urgent and emergency care and the prevalence of multimorbidity, in addition to other variables and instruments of interest. The prospective cohort design intends to estimate the risk of using and reusing urgent emergency services after 12 months. Contact information, collected to ensure follow-up, included telephone, social networks, and full address. In addition, we also collected the latitude and longitude of households for control of the interviews.

### Study location and target population

**Figura 1.**
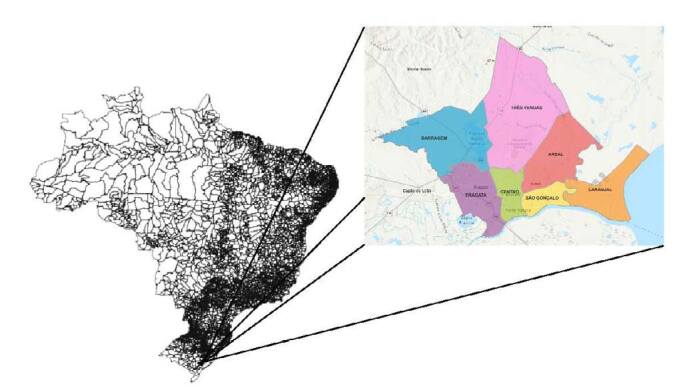
Map of Brazil highlighting the city of Pelotas (RS).

Source: https://pmpel.maps.arcgis.com/apps/View/index.html?appid=805046dd7e72460ea073579e20a75fcd

The present study was conducted in adult households in the Pelotas, Rio Grande do Sul (RS), Southern Brazil. According to estimates by the Brazilian Institute of Geography and Statistics (IBGE) in 2020, Pelotas had an estimated population of 343,132 individuals (https://cidades.ibge.gov.br/brasil/rs/pelotas/panorama). Figure 1 shows the location of the city of Pelotas in Brazil. 

Pelotas has a human development index (HDI) of 0.739 and a gross domestic product per capita (GDP) of BRL 27,586.96 (https://www.ibge.gov.br/cidades-e-estados/rs/pelotas.html). The municipality has a Municipal Emergency Room that operates 24 hours a day, seven days a week, and serves about 300 patients a day, according to data provided by the unit.

### Criteria for inclusion and exclusion of study participants

We included adults aged 18 years or older residing in the urban area of Pelotas. Children and individuals who were mentally unable to answer the questionnaire were not included in the sample.

### Sample calculation, sampling process, and data collection

The sample size was calculated considering three objectives. First, to determine the sample size required to assess the prevalence of urgent and emergency services use, it was considered an estimated prevalence of 9%, with±two percentage points as a margin of error and a 95% confidence level^13^, concluding that 785 individuals would be necessary. Second, for multimorbidity prevalence, an estimated prevalence of 25%, with ± three percentage points as a margin of error and a confidence level of 95% was used ^14,15^; reaching again, a total of 785 individuals needed. Finally, for the association calculations, similar studies in Brazil were assessed, and the following parameters were considered: significance level of 95%, power of 80%, exposed/unexposed ratio of 0.1, percentage of the outcome in the unexposed 20%, and a minimum prevalence ratio of 1.3. With these parameters, 5,104 individuals would be necessary to study the proposed associations. Adding 10 to 20% for losses and/or refusals, the final sample size would be composed of 5,615–5,890 participants.

The process to provide a population-based sample was carried out in multiple stages. The city of Pelotas has approximately 550 census tracts, according to the last update estimates provided by IBGE in 2019. From there, we randomly selected 100 sectors. Since the sectors vary in size, we defined a proportional number of households for each.

Thus, it was estimated that, in total, the 100 sectors had approximately 24,345 eligible households. To interview one resident per household, we divided the total number of households by the sample size required, which resulted in 4.3. Based on this information, we divided each of the 100 sectors by 4.3 to reach the necessary number of households for each sector. One resident per household was interviewed, resulting in a total of 5,615 households. If there was more than one eligible resident, the choice was made by a random number generator application. Residents were placed in order, a number was assigned for each one, and one of them was selected according to the result of the draw. The first household interviewed in each sector was selected through a draw, considering the selected jump (4.3 households). Trades and empty squares were considered ineligible, and thus, the next square was chosen. Due to a large number of empty houses, it was necessary to select another 50 sectors to complete the required sample size. The additional households were drawn according to the same methodological criteria as the first draw to ensure equiprobability.

### Data collection instrument

We collected the data with the Research Electronic Data Capture (REDCap), a data collection program using smartphones^16,17^. Experienced and trained research assistants collected the data. The questionnaire from EAI PELOTAS was prepared, when possible, based on standardized instruments, including questions about chronic diseases, physical activity, food security, use of urgent and emergency services, functional disability, frailty syndrome, self-perception of health, COVID-19, in addition to sociodemographic and behavioral questions. Supplementary Table 1 shows the instruments utilized in the present study.

**Table 1.** First descriptive results and comparison with a population-based study.

Characteristics	EAI PELOTAS*		PNS 2019^†^
	Crude % (95%CI)	Survey design % (95%CI)	% (95%CI)
Mean age, years	50.3 (49.9–50.8)	46.2 (45.5–47.0)	46.7 (45.9–47.5)
Mean number of household people	2.6 (2.5–2.7)	2.7 (2.6–2.8)	3.0 (2.9–3.1)
Female (%)	66.8 (65.6–68.0)	54.2 (52.4–55.6)	54.1 (51.7–56.4)
Skin color (%)			
White	78.2 (77.1–79.2)	77.3 (74.9–79.5)	76.8 (74.6–78.7)
Black	15.0 (14.1–16.0)	15.3 (13.5–17.3)	8.3 (7.0–9.8)
Brown	6.1 (5.5–6.7)	6.7 (5.7–7.9)	14.5 (12.9–16.3)
Other	0.7 (0.5–1.0)	0.7 (0.4–1.1)	0.4 (0.2–0.8)
Schooling (%)			
Incomplete elementary school or less	35.7 (34.5–37.0)	31.3 (28.6–34.2)	30.2 (28.1–32.4)
Complete elementary school/incomplete high school	16.2 (15.3–17.2)	16.4 (15.1–17.7)	15.7 (14.0–17.5)
Complete high school/incomplete higher education	33.5 (32.3–34.7)	37.6 (35.6–39.6)	36.9 (34.6–39.2)
Complete higher education or more	14.6 (13.7–15.5)	14.7 (12.4–17.4)	17.2 (15.7–18.9)

*n=5.722; ^†^n=3.002. PNS: Brazilian National Health Survey. PNS 2019 includes residents (selected to interview) from the urban area from the Rio Grande do Sul State; Survey design: weighted for primary unit sampling and post-weight estimates

### Dependent variables

The use of urgent and emergency services was assessed on a baseline using the following question: “In the last 12 months, how many times have you sought urgent and emergency services, such as an emergency room?”. This was followed by the characterization of the service used, city of service, frequency of use, and referral after use. One year after the study baseline, we will contact again the respondents to inquire about the use of urgent and emergency care services (number of times and type of service used). 

### Independent variables

We assessed multimorbidity as the main exposure using a list of 22 chronic diseases and others (asthma/bronchitis, osteoporosis, arthritis/arthrosis/rheumatism, hypertension, diabetes, cardiac insufficiency, pulmonary emphysema/chronic obstructive pulmonary disease, acute kidney failure, Parkinson’s disease, prostate disease, hypo/hyperthyroidism, glaucoma, cataract, Alzheimer’s disease, urinary/fecal incontinence, angina, stroke, dyslipidemia, epileptic fit/seizures, depression, gastric ulcer, urinary infection, pneumonia, and the flu). The association with urgent and emergency services will be performed with different cutoff points, including total number, ≥2, ≥3, and combinations of morbidities. We will also perform network analyzes to assess the pattern of morbidities.

Other independent variables were selected from previous studies in the literature^18-21^, including demographic, socioeconomic information, behavioral characteristics, health status, access, use and quality of health services.

### Data analysis

We will test artificial intelligence algorithms, ML, to predict the use of urgent and emergency services after 12 months. The purpose of ML is to predict health outcomes through the basic characteristics of the individuals, such as sex, education, and lifestyle. The algorithms will be trained to predict the occurrence of health outcomes, which will contribute to decision-making. With a good amount of data and the right algorithms, ML may be able to predict health outcomes with satisfactory performance. 

The area of ML in healthcare has shown rapid growth in recent years, having been used in significant public health problems such as diagnosing diseases and predicting the risk of adverse health events and deaths^22-24^. The use of predictive algorithms aims to improve health care and support decision-making by health professionals and managers. For the present study, individuals’ baseline characteristics will be used to train popular ML algorithms such as Support Vector Machine (SVM), Neural Networks (ANNs), Random Forests, Penalized Regressions, Gradient Boosted Trees, and Extreme Gradient Boosting (XGBoost). These models were chosen based on a previous review in which the authors identified the most used models in healthcare studies^25^. We will use the Python programming language to perform the analyzes.

To test the predictive performance of the algorithms in new unseen data, individuals will be divided into training (70% of patients, which will be used to define the parameters and hyperparameters of each algorithm) and testing (30%, which will be used to test the predictive ability of models in new data).

We will also perform all the preliminary steps to ensure a good performance of the algorithms, especially those related to the pre-processing of predictor variables, such as the standardization of continuous variables, separation of categorical predictors with one-hot encoding, exclusion of strongly correlated variables, dimension reduction using principal component analysis and selection of hyperparameters with 10-fold cross-validation. Different metrics will evaluate the predictive capacity of the models, the main one being the area under the receiver operating characteristic (ROC) curve (AUC). In a simplified way, the AUC is a value that varies from 0 to 1, and the closer to 1 the better the model’s predictive capacity^26^. The other metrics will be F1-score, sensitivity, specificity, and accuracy. As measures of model fit, we will perform hyperparameters and balancing fit, as well as K-fold (cross-validation).

## COVID-19

The current pandemic, caused by the SARS-CoV-2 virus, has brought uncertainty to the world population. Although vaccination coverage is already high in large parts of the population, the arrival of new variants and the lack of other essential measures to face the pandemic still create uncertainty about the effects of the pandemic on people. General questions about symptoms, tests, and possible effects caused by coronavirus contamination were included in our baseline survey. We will also use SARS-CoV-2-related questions to evaluate the performance of ML algorithms. In September 2021, restrictive measures were relaxed due to a decrease in COVID-19 cases in Pelotas, allowing the study to begin. A vaccination passport was required from the interviewers to ensure the safety of both participants and interviewers. In addition, all interviewers received protective equipment against COVID-19, including masks, face shields, and alcohol gel. Finally, the interviewers were instructed to conduct the research in an open and airy area, ensuring the protection of the participants.

### Quality assurance and control

The activities to allow for control and data quality were characterized by a series of measures aimed at ensuring results without the risk of bias. Initially, we developed a research protocol, followed by an instruction manual for each interviewer. Thereafter, interviewers were trained and standardized in all necessary aspects.

REDCap was also important to garanteee the control and quality of responses as the questions were designed using validation checks according to what was expected for each answer. Another measure that ensured the control of interviews was the collection of latitude and longitude of households, which was plotted by two members of the study coordination weekly on maps, to ensure that the data collection was performed according to the study sample. With latitude and longitude data, it is also intended to carry out spatial analysis articles with techniques such as sweep statistics and Kernel.

The database of the questions was checked daily to find possible inconsistencies. Finally, two members of the study coordination made random phone calls to 10% of the sample, in which a reduced questionnaire was applied, with the objective of comparing the answers with the main questionnaire.

### Ethical principles

We carried out this study using free and informed consent, as determined by the ethical aspects of Resolution No. 466/2012 of the National Council of the Ministry of Health and the Code of Ethics for Nursing Professionals, of the duties in Chapter IV, Article 35, 36 and 37, and the prohibitions in chapter V, article 53 and 54. After identifying and selecting the study participants, they were informed about the research objectives and signed the Informed Consent Form (ICF). The project was referred to the Research Ethics Committee via the Brazilian platform and approved under the CAAE 39096720.0.0000.5317.

### Schedule

Initially, we conducted a stage for the preparation of an electronic questionnaire at the beginning of 2021. In February 2021, we initiated data collection after preparing the online questionnaire. The database verification and cleaning steps occurred simultaneously with the collection, and continued until March 2022. After this step, data analysis and writing of scientific articles began.

## RESULTS

### First descriptive results and comparison with a population-based study

**Figura 2.**
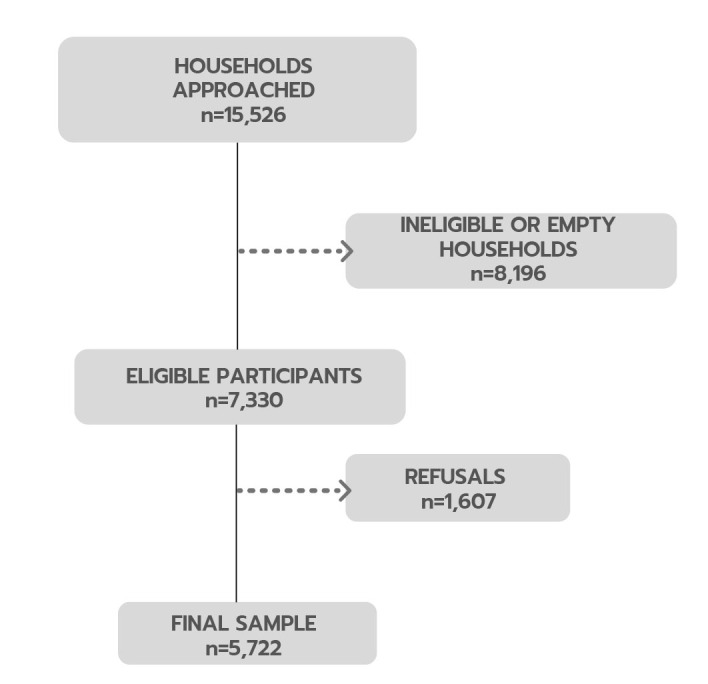
Flowchart describing the sampling process.

Of approximately 15,526 households approached, 8,196 were excluded — 4,761 residents were absent at the visit, 1,735 were ineligible, and 1,700 were empty (see Figure 2). We identified 7,330 eligible participants, of which 1,607 refused to participate in the study, totalizing 5,722 residents. Comparing the female gender percentage of the refusals with the completed interviews, we observed a slightly lower prevalence with 63.2% (95%CI 60.7–65.5) among the refusals, and 66.8% (95%CI 65.6–68.0) among the complete interviews. The mean age was similar between participants who agreed to participate (50.3; 95%CI 49.9–50.8) and those who refused (50.4; 95%CI 49.0–51.9).

To evaluate the first descriptive results of our sample, we compared our results with the 2019 Brazilian National Health Survey (PNS) database. The PNS 2019 was collected by the IBGE in partnership with the Ministry of Health. The data are in the public domain and are available in the IBGE website (https://www.ibge.gov.br/). To ensure the greatest possible comparability between studies, we used only residents of the urban area of the state of Rio Grande do Sul, aged using the command *svy* from Stata, resulting in 3,002 individuals (residents selected to interview).

We developed two models to compare our data with the PNS 2019 survey:

1. Crude model (crude results from the EAI PELOTAS study, without considering survey design estimates);
2. Model 1 using survey design: primary sampling units (PSUs) using census tracts as variables and post-weight variables based on estimates of Pelotas population projection for 2020 (Table 1). We evaluated another model using individual sampling weight (i.e., the inverse of the probability of being interviewed in each census tract). These models are virtually equal to the above estimates (data not shown).

The mean age of our sample was 50.3 years (Table 1), 46.2 for model 1, which was similar to PNS 2019 (46.7 years). Our weighted estimates presented a similar proportion of females compared to the PNS 2019 sample. The proportions of skin colors were similar in all categories and models. Our crude model presented a higher proportion of participants with incomplete elementary school or less compared to model 1 and PNS 2019.

Table 2 describes the prevalence of chronic diseases and lifestyle factors in our study and the PNS 2019 sample. Our prevalence of diabetes was higher in the crude model compared to weighted estimates and PNS 2019 sample. In both models, we had a higher proportion of individuals with obesity and hypertension than in PNS 2019. Asthma and/or bronchitis presented similar proportions in our results compared to PNS 2019; the same occurred for cancer. Our study presented a higher proportion of smoking participants in both models than in the PNS 2019 sample.

**Table 2.** First descriptive results and comparison with a population-based study.

Chronic diseases and lifestyle factors	EAI PELOTAS*		PNS 2019^†^
	Crude	Survey design 1	
	% (95%CI)	% (95%CI)	% (95%CI)
Diabetes	14.2 (13.3–15.1)	11.5 (10.6–12.4)	9.0 (8.9–11.1)
Obesity	30.4 (29.2–31.7)	29.2 (27.7–30.8)	24.8 (22.6–27.1)
Hypertension	39.0 (37.7–40.3)	32.4 (31.0–33.9)	28.1 (25.9–30.5)
Asthma or chronic bronchitis	9.3 (8.6–10.1)	9.3 (8.4–10.4)	8.7 (7.3–10.3)
Cancer	4.2 (3.7–4.7)	3.4 (2.9–4.0)	3.8 (2.9–4.9)
Current smoking	20.6 (19.6–21.7)	20.4 (18.9–22.0)	16.3 (14.6–18.1)

*n=5.722; ^†^n=3.002. PNS: Brazilian National Health Survey. PNS 2019 includes residents (selected to interview) from the urban area from the Rio Grande do Sul State; Survey design 1: weighted for primary unit sampling and post-weight estimates.

## DISCUSSION

We described the initial descriptive results, methodology, protocol, and the steps required to perform the ML analysis for predicting the use of urgent and emergency services among the residents of Pelotas, Southern Brazil. We expect to provide subsidies to health professionals and managers for decision-making, helping to identify interventions targeted at patients more likely to use urgent and emergency services, as well as those more likely to develop multimorbidity and mortality. We also expect to help health systems optimize their space and resources by directing human and physical capital to those at greater risk of developing multiple chronic diseases and dying. Recent studies in developed countries have found this a feasible challenge with ML^21,27^. If our study presents satisfactory results, we intend to test its practical applicability and acceptance to assist health professionals and managers in decision-making in emergency services among residents of Pelotas.

The baseline and methods used to select households resemble the main population-based studies conducted in Brazil, such as the Brazilian Longitudinal Study of Aging (ELSI-Brazil)^28^, the EPICOVID^29^, and the PNS. The applicability of ML requires suitable predictive variables. Our study included sociodemographic and behavioral variables related to urgent and emergency services, and chronic diseases. EAI PELOTAS study also includes essential topics that deserve particular importance during the COVID-19 pandemic, such as food insecurity, decreased income, physical activity, access to health services, and social support.

We also presented one weighting option in order to obtain sample estimates considering the complex study design. All estimates have their strength and limitation. Each research question answered through this study may consider these possibilities and choose the most suitable one. The estimates were similar without weighting and those considering the primary sampling unit (PSU) and sampling weight. Using the census tract in the PSU is fundamental to consider the sampling design in the estimates of variability (standard error, variance, 95%CI, among others). In addition, due to the possible selection bias in the sample, which contains more women and older people than expected, the use of a post-weighting strategy becomes necessary to obtain estimates adjusted for the sex and age distributions of the target population (due to the lack of census data, we used population projections). However, it should be noted that this strategy can produce estimates simulating the expected distribution only by sex and age. Still, we do not know how much this strategy can distort the estimates since the demographic adjustment cannot reproduce adjustment in all sample characteristics, especially for non-measured variables that may have influenced the selection of participants. Thus, we recommend defining the use of each strategy on a case-by-case basis, depending on the objective of the scientific product. Finally, we suggest reporting the different estimates according to the sample design for specific outcomes (e.g., the prevalence of a specific condition) that aim to extrapolate the data to the target population (adults of the city of Pelotas). 

In conclusion, the present article presented a protocol describing the steps that were and will be taken to produce a model capable of predicting the demand for urgent and emergency services in one year among residents in Pelotas (RS), Southern Brazil.

## SUPPLEMENTARY DATA:

Supplementary data are available at IJE online.
